# Serum Glycoprotein VI in Acute Pulmonary Embolism: Association With Radiological Thrombus Burden and Clinical Risk Indicators

**DOI:** 10.1155/carj/9286221

**Published:** 2026-10-18

**Authors:** Mert Metin İnan, Mustafa Burak Sayhan, Ömer Salt, Eray Çeliktürk, Sinem Çalın, Esin Seçgin Sayhan

**Affiliations:** ^1^ Department of Emergency Medicine Trakya University Medicine Faculty Edirne Turkey; ^2^ Emergency Department Kayseri Training and Research Hospital Edirne Turkey saglik.gov.tr; ^3^ Department of Medical Biochemistry Trakya University Medicine Faculty Edirne Turkey; ^4^ Department of Public Health Trakya University Medicine Faculty Edirne Turkey trjums.ac.ir

**Keywords:** biomarkers, platelet activation, platelet membrane glycoproteins, pulmonary embolism, risk assessment

## Abstract

**Background:**

Pulmonary embolism (PE) is a life‐threatening condition requiring rapid diagnosis and accurate risk stratification. Biomarkers reflecting thromboinflammatory processes may improve early clinical assessment. Glycoprotein VI (GPVI), a key receptor involved in platelet activation, has been implicated in thrombus formation; however, its clinical relevance in PE remains unclear.

**Objective:**

To examine the association of serum GPVI with radiological thrombus burden and clinical risk indicators in acute PE, while retaining the original PE‐versus‐control comparison only as a secondary exploratory case–control discrimination analysis, not as a diagnostic‐accuracy analysis.

**Methods:**

In this cross‐sectional study, the analytic cohort comprised 50 patients with CTPA‐confirmed acute PE and 25 control participants. Serum soluble GPVI (sGPVI) was measured by a colorimetric sandwich ELISA. The study‐recorded CTPA thrombus‐burden categories were analyzed as lower, intermediate, and higher radiological burden and separately from an exploratory ESC‐aligned risk reconstruction. For ICU admission, parsimonious multivariable and incremental analyses evaluated GPVI beyond PESI with internal validation. A PE‐versus‐control ROC comparison with D‐dimer was retained only as a secondary exploratory case–control discrimination analysis and was not treated as an estimate of diagnostic accuracy.

**Results:**

Among patients with PE, GPVI differed across the study‐recorded radiological burden categories (lower, *n* = 16: 192.95 [185.80–215.39]; intermediate, *n* = 13: 258.54 [238.60–276.29]; higher, *n* = 21: 307.90 [285.92–321.95] ng/L; *p* < 0.001). In the exploratory ESC‐aligned analysis, GPVI differed across risk categories (Kruskal–Wallis *p* = 0.045) and showed an ordinal association with increasing risk category (Spearman *ρ* = 0.331, *p* = 0.02). GPVI was higher in patients admitted to the ICU than in those not admitted (298.24 [278.42–315.89] vs. 206.17 [191.15–238.33] ng/L, *p* < 0.001) and had an ICU‐admission AUC of 0.969. In a parsimonious model adjusted for PESI, GPVI remained associated with ICU admission (OR per 10 ng/L, 2.31; 95% CI, 1.36–3.92; *p* = 0.002), although the magnitude should be interpreted cautiously given the small sample size, limited number of ICU events, and strong separation. These effect estimates are exploratory and should not be interpreted as stable clinical effect sizes. Adding GPVI to PESI increased the apparent AUC from 0.885 to 0.968 (ΔAUC, 0.083; bootstrap 95% CI, 0.013–0.200); the optimism‐corrected AUC was approximately 0.954, and repeated stratified 5‐fold cross‐validation yielded a mean AUC of approximately 0.916. In the secondary PE‐versus‐control analysis, GPVI was higher in PE (268.12 [215.39–298.47] vs. 114.07 [89.10–136.80] ng/L, *p* < 0.001) and showed an AUC of 0.968 versus 0.884 for D‐dimer (ΔAUC, 0.084; bootstrap 95% CI, 0.008–0.168; *p* = 0.03); this case–control estimate is susceptible to spectrum bias and is not an estimate of real‐world diagnostic accuracy.

**Conclusion:**

Serum GPVI was associated with radiological thrombus burden and severity‐related clinical indicators in acute PE. These exploratory findings support further evaluation of GPVI as a potential risk‐stratification biomarker but do not establish diagnostic utility. Confirmation in larger, clinically representative prospective cohorts with hard clinical outcomes is required.

## 1. Introduction

Pulmonary embolism (PE) represents an acute and potentially life‐threatening manifestation of venous thromboembolism, in which early diagnosis and appropriate risk stratification are essential for reducing mortality [1]. The wide spectrum of clinical presentation, ranging from hemodynamically stable cases to fulminant forms, underscores the need for reliable early risk markers [1–3]. Current guidelines recommend the combined use of clinical scoring systems and biomarkers in the management of PE [1, 3, 4]. However, most existing risk stratification tools rely predominantly on clinical parameters and markers of cardiac injury, while biomarkers that directly reflect the underlying biological mechanisms of thrombosis remain limited. PE cannot be explained solely by activation of the coagulation cascade, as its pathophysiology involves a complex interplay of platelet activation, inflammatory response, and thrombus stabilization, reflecting a thromboinflammatory process [5]. Accordingly, biomarkers related to platelet activation have been increasingly investigated in recent years as potential indicators of disease severity and clinical outcomes in PE. Glycoprotein VI (GPVI), a receptor expressed on the platelet surface, plays a central role in collagen‐mediated platelet activation, platelet adhesion, and thrombus stabilization. Experimental and translational studies have demonstrated that GPVI is an important regulator of thrombus formation and thrombus progression [6–8]. However, the clinical role of GPVI in the context of venous thrombosis, particularly PE, has not yet been sufficiently elucidated [9]. Clinical biomarker work on GPVI has concentrated largely in arterial disease such as acute coronary syndrome [10], while PE studies have more often focused on inflammatory indices and cardiac biomarkers [5, 11]. This gap highlights the need for further exploration of biomarkers that more accurately represent the biological mechanisms underlying thrombosis.

In this study, we examined associations between serum GPVI and study‐recorded radiological thrombus burden, guideline‐aligned clinical risk indicators, and ICU admission among patients with computed tomography pulmonary angiography (CTPA)–confirmed acute PE and explored whether GPVI provided severity‐related information beyond PESI. The original PE‐versus‐control comparison was retained only as a secondary exploratory case–control analysis.

## 2. Materials and Methods

### 2.1. Study Design and Population

This single‐center cross‐sectional study was conducted in the emergency department of a tertiary university hospital between January 2023 and June 2023. The analyzed cohort comprised 75 participants: 50 patients evaluated for suspected acute PE in whom PE was confirmed by clinically indicated CTPA and 25 control participants (33.3% of the cohort) who presented to the same emergency department during the same period with a variety of other acute complaints and in whom PE was not clinically suspected. Because PE was not clinically suspected in the control participants, CTPA was not indicated and was not performed; PE was therefore not formally excluded by the reference standard in this group. Control participants were emergency department attendees rather than healthy volunteers, and they do not represent the symptomatic suspected‐PE population in whom a diagnostic test would be applied in practice. No symptomatic CTPA‐negative comparator group was included in the analyzed cohort. Each participant was evaluated on a single occasion at enrollment; there were no repeated GPVI measurements or protocol‐specified longitudinal study visits. The study laboratory panel, including D‐dimer, troponin, B‐type natriuretic peptide, and lactate, was obtained during that single enrollment assessment following written informed consent. The same exclusion criteria were applied to both groups, and ethics committee approval covered enrollment of both groups. Chronic or recurrent PE; active malignancy or malignancy within the past 5 years; atrial fibrillation; active infection; autoimmune or chronic inflammatory disease; major surgery or trauma within 3 months; immunosuppressive therapy; pregnancy; missing clinical or laboratory data; and lack of written informed consent were exclusion criteria. The participant flow is summarized in Figure 1.

**FIGURE 1:**
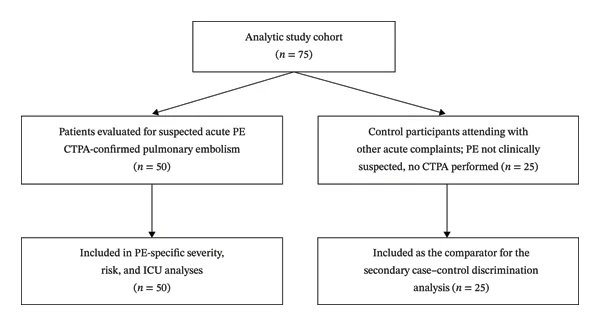
Participant flow diagram. The analytic cohort comprised 75 participants: 50 patients with CTPA‐confirmed acute PE and 25 control participants presenting to the same emergency department with other acute complaints in whom PE was not clinically suspected. Because PE was not suspected, CTPA was not indicated in the control group, and PE was not formally excluded by the reference standard in these participants. The PE group contributed to all severity, risk, and ICU analyses; control participants served only as the comparator for the secondary case–control discrimination analysis. CTPA: computed tomography pulmonary angiography; PE: pulmonary embolism.

No formal a priori sample‐size calculation was performed because this was an exploratory biomarker study enrolling eligible patients during a predefined study period. In view of the modest sample and event counts, multivariable analyses were deliberately parsimonious and were accompanied by internal validation.

### 2.2. Clinical Severity Assessment

For patients with confirmed PE, the original case‐report form recorded an ordinal CTPA‐based thrombus‐burden category using three operational definitions: Category 1, originally termed “severe PE,” indicated pulmonary artery embolism; Category 2, originally termed “moderate PE,” indicated pulmonary artery branch embolism; and Category 3, originally termed “mild PE,” indicated a single pulmonary arteriole embolism. Because the terms mild, moderate, and severe could be confused with guideline‐defined clinical risk, the revised manuscript relabels these study‐recorded categories as higher, intermediate, and lower radiological burden, respectively. This legacy CRF classification is an anatomical/radiological descriptor and is not a validated clinical risk score. CTPA‐based clot‐burden quantification has precedent in the literature, but the study‐recorded three‐level scheme is not equivalent to validated obstruction indices such as the Qanadli index [12]. The qSOFA score was calculated only as an exploratory measure of acute physiological derangement; qSOFA is not a PE‐specific guideline risk score and was not used in the ESC‐aligned classification.

For complementary guideline‐based clinical risk assessment, an exploratory ESC‐aligned risk classification was reconstructed from the baseline variables available in the dataset. Right‐ventricular dysfunction was defined as a CTPA RV/LV ratio ≥ 1.0, and cardiac biomarker positivity was defined as troponin > 19.8 ng/L according to the local laboratory upper reference limit. Patients with admission systolic blood pressure < 90 mmHg were flagged as a possible high‐risk proxy; because persistence of hypotension, vasopressor requirement, obstructive shock, and cardiac arrest were not systematically recorded, this proxy was not considered equivalent to formally documented ESC high‐risk PE. Among the remaining patients, intermediate‐high risk required PESI > 85 together with RV dysfunction and troponin elevation; low risk required PESI ≤ 85 with neither RV dysfunction nor troponin elevation; all remaining patients were classified as intermediate‐low risk for this exploratory reconstruction.

### 2.3. Soluble Glycoprotein VI (sGPVI) Measurement

Blood samples (3 mL) were obtained during the emergency‐department evaluation and collected into standard biochemistry tubes. The precise interval from symptom onset to sampling and the temporal relationship between sampling and anticoagulant administration were not prospectively recorded. Samples were allowed to clot at room temperature for 120 min and were subsequently centrifuged at 1000 rpm for 20 min using a refrigerated centrifuge maintained at +4°C.

Following centrifugation, the serum fraction was carefully separated using a pipette and transferred into Eppendorf tubes. The aliquoted serum samples were stored at −20°C until the day of analysis.

Serum sGPVI concentrations were measured in the Department of Medical Biology using a commercially available Human GPVI (platelet GPVI) ELISA kit (Catalog No. E4824Hu, BTLAB Co., China). The assay is a colorimetric sandwich ELISA specified for human serum. Because the analyte was quantified in cell‐free serum, the reported concentration represents circulating sGPVI rather than platelet‐surface receptor expression; the abbreviation GPVI is retained in the tables and figures for consistency with the source dataset. The supplier‐specified detection range was 30–7000 ng/L, and the detection limit was 16.39 ng/L.

Absorbance measurements were performed using a Thermo Fisher Scientific Multiskan GO microplate reader (model 1510‐03837), and concentrations were derived according to the manufacturer’s recommended assay protocol.

### 2.4. Ethics Approval and Funding

The study protocol was reviewed and approved by the Trakya University Ethics Committee (Approval No: [TUTF‐GOBAEK 2022/406, Approval Date: 12 December 2022]) and was conducted in accordance with the principles of the Declaration of Helsinki. Written informed consent was obtained from all participants or their legal representatives prior to enrollment. No financial support or external funding was received for this study.

### 2.5. Statistical Analysis

Continuous variables were assessed using visual inspection and normality testing and are reported as median (interquartile range [IQR]) because the principal continuous variables were non‐normally distributed. Categorical variables are presented as number and percentage. Two independent groups were compared using the Mann–Whitney *U* test, and more than two groups using the Kruskal–Wallis test. Categorical variables were compared using the chi‐square test. Associations between GPVI and D‐dimer, troponin, neutrophil‐to‐lymphocyte ratio (NLR), systemic immune‐inflammation index (SII), lactate, and ordinal ESC‐aligned risk category were evaluated using two‐sided Spearman rank correlation coefficients (*ρ*).

ROC analysis quantified discrimination for CTPA‐confirmed PE versus control participants, the higher CTPA‐based radiological burden category versus the two lower burden categories, and ICU admission. AUCs are reported with 95% confidence intervals (CIs), and marker‐specific optimal cutoffs were selected using the Youden index. Within the original PE‐versus‐control design, GPVI and D‐dimer AUCs were compared using stratified bootstrap resampling (10,000 replicates). Because no symptomatic CTPA‐negative comparator group was available in the analyzed cohort, this diagnostic ROC analysis was treated as exploratory case–control discrimination and not as an estimate of real‐world diagnostic accuracy.

For ICU admission, the primary parsimonious multivariable model included GPVI (scaled per 10 ng/L) and PESI (scaled per 10 points). To address potential confounding without overparameterizing the model, sensitivity models evaluated GPVI after separate adjustment for age, troponin, BNP, lactate, NLR, SII, and D‐dimer. Exploratory incremental discrimination was restricted to PESI alone versus PESI + GPVI. Change in AUC, integrated discrimination improvement (IDI), and category‐free net reclassification improvement (NRI) were estimated using 10,000 outcome‐stratified nonparametric bootstrap resamples with model refitting; percentile 95% CIs were used, and two‐sided *p* values for IDI and NRI were obtained from the estimate divided by its bootstrap standard error using a normal approximation. Category‐free NRI was the sum of event and nonevent net reclassification components, and IDI was the difference between the two models’ discrimination slopes. Internal validation was performed for both PESI and PESI + GPVI. Optimism‐corrected AUC and Brier score used 2000 ordinary nonparametric bootstrap resamples with model refitting. Repeated stratified 5‐fold cross‐validation used 200 repeats (1000 test folds); out‐of‐fold probabilities were pooled within each repeat, and the mean, standard deviation, and empirical 2.5th–97.5th percentile range of the repeat‐level AUCs were reported. These empirical ranges summarize variability across repeated random data partitions and are not CIs. A fixed random seed (20260820) was used for the final resampling analyses. Conventional descriptive statistics, group comparisons, correlations, and regression analyses were performed using IBM SPSS Statistics for Windows, Version 25.0 (IBM Corp., Armonk, NY, USA). Advanced ROC, resampling, internal‐validation, and reclassification analyses were performed in R Version 4.6.1 (R Foundation for Statistical Computing, Vienna, Austria), using pROC Version 1.19.0.1 and rms Version 8.1‐1; IDI and NRI were calculated directly from paired model‐predicted probabilities with custom resampling code. All tests were two‐sided with *α* = 0.05. *p* values are reported according to the journal’s requested conventions; the borderline ESC‐category comparison is reported to three decimals (*p* = 0.045) to avoid ambiguity.

## 3. Results

### 3.1. Study Population and Baseline Characteristics

A total of 50 patients with CTPA‐confirmed PE and 25 control participants were included. The groups did not differ significantly in age or sex distribution (*p* > 0.05). White blood cell count, NLR, SII, D‐dimer, troponin, BNP, and lactate were higher in the PE group, whereas platelet counts were similar between groups. Serum GPVI was substantially higher in the PE group (268.12 [215.39–298.47] vs. 114.07 [89.10–136.80] ng/L, *p* < 0.001) (Table 1, Figure 2A). The control participants were not a symptomatic suspected‐PE comparator group.

**TABLE 1:** Baseline demographic and laboratory characteristics of patients with pulmonary embolism and control participants.

Variable	Pulmonary embolism (*n* = 50)	Control participants (*n* = 25)	*p* value
Age (years)	70.5 (64.0–77.8)	68.0 (65.0–70.0)	0.16
Female sex, *n* (%)	23 (46)	14 (56)	0.41
WBC (× 10^9^/L)	11.4 (9.6–16.6)	7.6 (6.6–9.8)	< 0.001
Platelet (× 10^9^/L)	267 (193–396)	257 (225–307)	0.75
NLR (ratio)	7.50 (4.51–14.72)	3.17 (1.78–4.89)	< 0.001
SII (index)	1771 (1222–3453)	713 (417–1121)	< 0.001
D‐dimer (mg/L)	5.60 (1.25–9.88)	0.30 (0.20–0.79)	< 0.001
Troponin (ng/L)	62.8 (13.9–271.7)	2.5 (1.8–2.8)	< 0.001
BNP (pg/mL)	599 (380–2410)	55 (44–98)	< 0.001
Lactate (mg/dL)	19.5 (12.1–27.8)	9.0 (6.0–11.0)	< 0.001
GPVI (ng/L)	268.12 (215.39–298.47)	114.07 (89.10–136.80)	< 0.001

*Note:* Values are median (IQR) unless otherwise specified. Continuous variables were compared using the Mann–Whitney *U* test and sex distribution using the chi‐square test. GPVI: glycoprotein VI. Troponin is reported in ng/L and lactate in mg/dL, consistent with the verified patient‐level analysis dataset. Control participants were emergency department attendees in whom PE was not clinically suspected; CTPA was not indicated and was not performed.

Abbreviations: BNP, B‐type natriuretic peptide; NLR, neutrophil‐to‐lymphocyte ratio; SII, systemic immune‐inflammation index.

**FIGURE 2:**
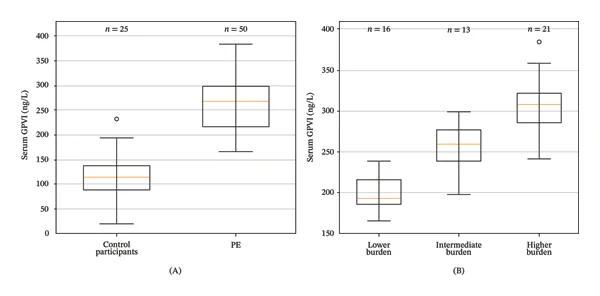
Serum glycoprotein VI (GPVI) according to PE status and study‐recorded CTPA‐based radiological thrombus burden. (A) Patient‐level boxplots of serum GPVI in patients with CTPA‐confirmed PE and control participants. (B) Patient‐level boxplots of GPVI across lower‐, intermediate‐, and higher‐burden radiological categories. In the legacy case‐report form, lower burden corresponded to a single pulmonary arteriole embolism, intermediate burden to pulmonary artery branch embolism, and higher burden to pulmonary artery embolism; sample sizes are displayed for each category. CTPA: computed tomography pulmonary angiography; GPVI: glycoprotein VI; PE: pulmonary embolism. The radiological burden categories are study‐recorded descriptors and are not equivalent to ESC clinical risk categories.

### 3.2. GPVI Levels According to CTPA‐Based Radiological Thrombus Burden

GPVI differed across the study‐recorded ordinal CTPA‐based radiological thrombus‐burden categories. Median GPVI was 192.95 (185.80–215.39) ng/L in the lower‐burden category (single pulmonary arteriole embolism; *n* = 16), 258.54 (238.60–276.29) ng/L in the intermediate‐burden category (pulmonary artery branch embolism; *n* = 13), and 307.90 (285.92–321.95) ng/L in the higher‐burden category (pulmonary artery embolism; *n* = 21) (Kruskal–Wallis *p* < 0.001) (Table 2, Figure 2B). These labels describe radiological burden only and should not be interpreted as guideline‐defined clinical severity.

**TABLE 2:** Serum GPVI according to study‐recorded CTPA‐based radiological thrombus‐burden categories.

Radiological burden category (legacy CRF definition)	*n*	GPVI, median (IQR) (ng/L)	*p* value
Lower (single pulmonary arteriole embolism)	16	192.95 (185.80–215.39)	< 0.001
Intermediate (pulmonary artery branch embolism)	13	258.54 (238.60–276.29)	
Higher (pulmonary artery embolism)	21	307.90 (285.92–321.95)	

*Note:* Comparisons across groups were performed using the Kruskal–Wallis test; the *p* value shown is the overall three‐group comparison.

### 3.3. Correlation of GPVI With Thrombotic Burden and Systemic Inflammation

Spearman rank correlation analysis did not identify significant monotonic associations between GPVI and the evaluated conventional thrombotic, cardiac, inflammatory, or perfusion markers. Correlations were weak for D‐dimer (*ρ* = 0.07, *p* = 0.63; Figure 3A), troponin (*ρ* = 0.09, *p* = 0.54; Figure 3B), NLR (*ρ* = −0.09, *p* = 0.54; Figure 3C), SII (*ρ* = 0.21, *p* = 0.15; Figure 3D), and lactate (*ρ* = 0.06, *p* = 0.70; Figure 3E) (Table 3).

**FIGURE 3:**
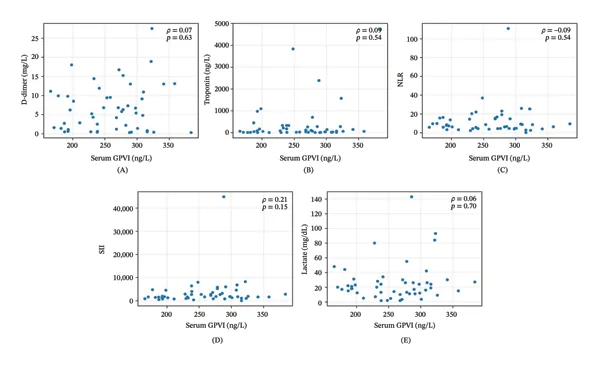
Relationships between serum GPVI and conventional laboratory markers in patients with pulmonary embolism. Scatter plots show GPVI in relation to (A) D‐dimer, (B) troponin, (C) neutrophil‐to‐lymphocyte ratio (NLR), (D) systemic immune‐inflammation index (SII), and (E) lactate. Spearman rank correlation coefficients (*ρ*) and corresponding two‐sided *p* values are displayed in each panel; none of the five correlations was statistically significant. GPVI: glycoprotein VI; NLR: neutrophil‐to‐lymphocyte ratio; SII: systemic immune‐inflammation index.

**TABLE 3:** Correlation of serum GPVI levels with markers of thrombotic burden and systemic inflammation.

Variable	*ρ*	*p* value
D‐dimer	0.07	0.63
Troponin	0.09	0.54
NLR (ratio)	−0.09	0.54
SII (index)	0.21	0.15
Lactate	0.06	0.70

*Note:* Correlations were assessed using two‐sided Spearman rank correlation analysis.

Abbreviations: NLR, neutrophil‐to‐lymphocyte ratio; SII, systemic immune‐inflammation index.

### 3.4. GPVI Levels According to Clinical Severity Indicators

GPVI was higher across several clinical severity indicators. Patients with qSOFA ≥ 2 had higher GPVI than those with qSOFA < 2 (308.44 [288.88–322.26] vs. 232.41 [192.86–265.73] ng/L, *p* < 0.001). GPVI was also higher in patients with PESI > 85 than in those with PESI ≤ 85 (287.18 [268.12–309.80] vs. 197.20 [187.31–228.61] ng/L, *p* < 0.001) and in patients admitted to the ICU compared with those not admitted (298.24 [278.42–315.89] vs. 206.17 [191.15–238.33] ng/L, *p* < 0.001) (Table 4). qSOFA was interpreted only as an exploratory physiological severity marker, not as a PE‐specific risk score.

**TABLE 4:** GPVI levels according to clinical severity indicators.

Variable	GPVI, median (IQR) (ng/L)	*p* value
qSOFA ≥ 2 (*n* = 20)	308.44 (288.88–322.26)	< 0.001
qSOFA < 2 (*n* = 30)	232.41 (192.86–265.73)	

PESI > 85 (*n* = 34)	287.18 (268.12–309.80)	< 0.001
PESI ≤ 85 (*n* = 16)	197.20 (187.31–228.61)	

ICU admission (*n* = 26)	298.24 (278.42–315.89)	< 0.001
No ICU admission (*n* = 24)	206.17 (191.15–238.33)	

*Note:* Comparisons between groups were performed using the Mann–Whitney *U* test.

### 3.5. Exploratory ESC‐Aligned Risk Analysis

The exploratory ESC‐aligned reconstruction classified 3 patients as low risk, 33 as intermediate‐low risk, 11 as intermediate‐high risk, and 3 as meeting the admission blood‐pressure proxy for possible high‐risk PE. Median GPVI values were 182.03 (176.53–187.57), 268.12 (211.10–298.70), 270.96 (244.86–305.19), and 297.77 (282.95–306.83) ng/L, respectively (Kruskal–Wallis *H* = 8.07, *p* = 0.045). Increasing ordinal risk category was associated with higher GPVI (Spearman *ρ* = 0.331, *p* = 0.02) (Table 5). Because the high‐risk category was reconstructed from a single admission systolic blood‐pressure value, it is presented as a proxy rather than as definitive guideline‐adjudicated high‐risk PE.

**TABLE 5:** Serum GPVI according to exploratory ESC‐aligned risk categories.

ESC‐aligned category	*n*	GPVI, median (IQR) (ng/L)	*p* value
Low	3	182.03 (176.53–187.57)	0.045
Intermediate‐low	33	268.12 (211.10–298.70)	
Intermediate‐high	11	270.96 (244.86–305.19)	
Possible high‐risk proxy^∗^	3	297.77 (282.95–306.83)	

^∗^Possible high‐risk proxy was defined by admission systolic blood pressure < 90 mmHg because persistent hypotension, shock, vasopressor requirement, and cardiac arrest were not systematically recorded. RV dysfunction: CTPA RV/LV ratio ≥ 1.0; troponin positivity: > 19.8 ng/L. The overall *p* value is from the Kruskal–Wallis test.

### 3.6. ROC Analyses

Within the PE cohort, GPVI discriminated the higher radiological burden category from the two lower categories with an AUC of 0.952 (95% CI, 0.880–0.980), at an optimal cutoff of 278.4 ng/L (sensitivity 85.7%, specificity 89.7%). For ICU admission, GPVI achieved an AUC of 0.969 (95% CI, 0.920–0.990), at an optimal cutoff of 271.0 ng/L (sensitivity 88.5%, specificity 95.8%). Both analyses were performed within the 50 patients with CTPA‐confirmed PE and describe within‐cohort discrimination for severity‐related endpoints; neither is externally validated.

### 3.7. Secondary Case–Control ROC Analysis

In the secondary comparison of CTPA‐confirmed PE with control participants, GPVI achieved an AUC of 0.968 (95% CI, 0.924–1.000), with an optimal cutoff of 171.02 ng/L (sensitivity 98.0%, specificity 88.0%), whereas D‐dimer achieved an AUC of 0.884 (95% CI, 0.801–0.950), with an optimal cutoff of 2.20 mg/L (sensitivity 68.0%, specificity 88.0%). The bootstrap‐estimated AUC difference was 0.084 (95% CI, 0.008–0.168; *p* = 0.03). Because the comparator group consisted of emergency department patients in whom PE was not clinically suspected and who therefore did not undergo CTPA, this analysis is subject to spectrum bias and does not estimate diagnostic accuracy in patients with suspected PE. It is reported only as a secondary exploratory finding (Table 6, Figure 4A).

**TABLE 6:** ROC analyses of GPVI for severity‐related endpoints within the PE cohort and secondary case–control discrimination of PE versus control participants.

Marker/outcome	AUC	95% CI	Cutoff	Sensitivity (%)	Specificity (%)	*p* value
GPVI: higher radiological burden category	0.952	0.880–0.980	278.4 ng/L	85.7	89.7	< 0.001
GPVI: ICU admission	0.969	0.920–0.990	271.0 ng/L	88.5	95.8	< 0.001
GPVI: PE vs. control participants	0.968	0.924–1.000	171.02 ng/L	98.0	88.0	< 0.001
D‐dimer: PE vs. control participants	0.884	0.801–0.950	2.20 mg/L	68.0	88.0	< 0.001

*Note:* AUC: area under the receiver operating characteristic curve; GPVI: glycoprotein VI. Marker‐specific optimal cutoffs were determined with the Youden index. The first two analyses were performed within the 50 patients with CTPA‐confirmed PE and describe within‐cohort discrimination for severity‐related endpoints. The PE‐versus‐control comparison is secondary and exploratory: control participants did not undergo CTPA, so this analysis is subject to spectrum bias and does not estimate diagnostic accuracy among patients with suspected PE. For the GPVI PE‐versus‐control AUC, the upper 95% confidence bound reached the theoretical maximum of 1.000.

Abbreviations: CI, confidence interval; ICU, intensive care unit; PE, pulmonary embolism.

**FIGURE 4:**
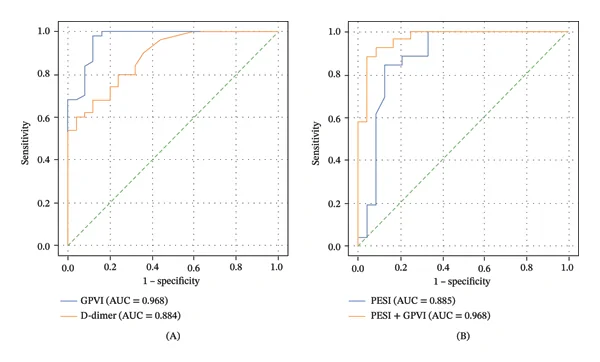
Receiver operating characteristic (ROC) analyses for secondary case–control discrimination and exploratory incremental ICU‐risk discrimination. (A) Secondary case–control discrimination of CTPA‐confirmed PE versus control participants by GPVI and by D‐dimer. (B) Discrimination of ICU admission within the PE cohort by PESI alone and by the combined PESI + GPVI model. The diagonal reference line represents chance discrimination. AUC values are displayed in the legends. (A) is a secondary exploratory analysis: control participants were emergency department patients in whom PE was not clinically suspected and who did not undergo CTPA, so (A) should not be interpreted as diagnostic accuracy in a suspected‐PE population. AUC: area under the curve; GPVI: glycoprotein VI; ICU: intensive care unit; PE: pulmonary embolism; PESI: pulmonary embolism severity index.

### 3.8. Parsimonious Multivariable Analysis for ICU Admission

Because the CTPA‐based radiological burden category was not a guideline‐defined clinical risk endpoint, the primary confounder‐adjusted analysis used ICU admission as the available severity‐related endpoint rather than the higher radiological burden category. In the model containing GPVI and PESI, each 10‐ng/L increase in GPVI was associated with higher odds of ICU admission (OR, 2.31; 95% CI, 1.36–3.92; *p* = 0.002), whereas PESI was not independently associated after GPVI adjustment (OR per 10 points, 1.02; 95% CI, 0.77–1.35; *p* = 0.90). In sensitivity models, GPVI remained statistically significant after separate adjustment for age, troponin, BNP, lactate, NLR, SII, and D‐dimer (Table 7). Because the sample was modest and GPVI showed strong separation across a limited number of ICU events, the relatively large ORs should be interpreted cautiously as exploratory estimates rather than as stable clinical effect sizes.

**TABLE 7:** Exploratory sensitivity analyses of the association between GPVI and ICU admission.

Adjustment model	GPVI OR per 10 ng/L	95% CI	*p* value	Adjustment covariate OR	95% CI	*p* value
PESI (per 10 points)	2.31	1.36–3.92	0.002	1.02	0.77–1.35	0.90
Age (per 10 years)	2.19	1.34–3.58	0.002	1.74	0.66–4.59	0.27
Troponin (per doubling)	5.62	1.29–24.49	0.02	2.87	0.94–8.79	0.07
BNP (per doubling)	2.27	1.39–3.70	0.001	1.17	0.63–2.17	0.63
Lactate (per doubling)	2.32	1.42–3.79	0.001	1.18	0.61–2.30	0.63
NLR (per doubling)	2.88	1.45–5.71	0.002	2.39	1.02–5.61	0.05
SII (per doubling)	2.92	1.40–6.12	0.004	2.04	1.02–4.11	0.05
D‐dimer (per doubling)	2.34	1.42–3.86	0.001	0.98	0.51–1.89	0.96

*Note:* Each model included GPVI and one covariate. GPVI is scaled per 10 ng/L; biomarkers are log_2_‐transformed where indicated. Large ORs and the wide troponin‐model CI reflect limited events/strong separation and should be interpreted cautiously.

Abbreviations: BNP, B‐type natriuretic peptide; CI, confidence interval; NLR, neutrophil‐to‐lymphocyte ratio; OR, odds ratio; SII, systemic immune‐inflammation index.

### 3.9. Exploratory Incremental Value and Internal Validation

PESI alone had an apparent AUC of 0.885 for ICU admission, and adding GPVI increased the apparent AUC to 0.968 (ΔAUC, 0.083; bootstrap 95% CI, 0.013–0.200); the likelihood‐ratio test for addition of GPVI was *p* < 0.001 (Figure 4B). With 2000 bootstrap resamples, optimism‐corrected AUCs were 0.885 for PESI and 0.954 for PESI + GPVI; corresponding apparent/optimism‐corrected Brier scores were 0.148/0.160 and 0.069/0.090. Across 200 repeats of stratified 5‐fold cross‐validation, the mean AUC was 0.863 (SD, 0.012) for PESI and 0.916 (SD, 0.020) for PESI + GPVI; the empirical 2.5th–97.5th percentile ranges across repeat‐level estimates were 0.837–0.882 and 0.867–0.939, respectively. The mean cross‐validated ΔAUC was 0.052, with an empirical 2.5th–97.5th percentile range of 0.002–0.093. These ranges describe variability across repeated random partitions and are not CIs. Secondary reclassification metrics were IDI = 0.367 (95% CI, 0.132–0.726; *p* = 0.02) and category‐free NRI = 1.532 (95% CI, 0.891–2.000; *p* < 0.001), with the upper percentile limit reaching the theoretical maximum of 2.000. Given the modest sample and the instability of reclassification measures in small datasets, these estimates are hypothesis‐generating and are not interpreted as evidence of established incremental clinical utility (Table 8).

**TABLE 8:** Exploratory incremental discrimination and internal validation of GPVI for ICU admission.

Model	Apparent AUC	Optimism‐corrected AUC	Repeated stratified 5‐fold CV AUC, mean (empirical 95% range)	Brier score, apparent/optimism‐corrected
PESI	0.885	0.885	0.863 (0.837–0.882)	0.148/0.160
PESI + GPVI	0.968	0.954	0.916 (0.867–0.939)	0.069/0.090

*Note:* Optimism correction used 2000 bootstrap resamples. Cross‐validation used 200 repeats; the displayed ranges are the empirical 2.5th–97.5th percentiles of repeat‐level AUCs and describe variation across repeated data partitions rather than confidence intervals. Lower Brier scores indicate lower prediction error. IDI and category‐free NRI are secondary exploratory metrics only; their uncertainty is reported in the text. The analysis is not an externally validated prediction model.

Abbreviations: AUC, area under the curve; CI, confidence interval; CV, cross‐validation; IDI, integrated discrimination improvement; NRI, net reclassification improvement.

## 4. Discussion

In this cross‐sectional study, the principal findings were severity‐related. Among patients with CTPA‐confirmed PE, serum GPVI differed across study‐recorded radiological burden categories, was higher with qSOFA ≥ 2, PESI > 85, and ICU admission, and showed an ordinal association with the exploratory ESC‐aligned risk reconstruction. GPVI remained associated with ICU admission after parsimonious adjustment for PESI, while incremental analyses were deliberately restricted and internally validated. In contrast, GPVI did not correlate significantly with D‐dimer, troponin, NLR, SII, or lactate. GPVI was also substantially higher in PE than in control participants, but the corresponding ROC analysis represents secondary case–control discrimination only; because no symptomatic CTPA‐negative comparator was available, it is vulnerable to spectrum bias and should not be interpreted as real‐world diagnostic accuracy. Overall, the findings are hypothesis‐generating associations rather than evidence of established clinical utility.

PE is an acute manifestation of venous thromboembolism, characterized by thrombus formation within the pulmonary arterial bed and the resulting hemodynamic impairment [1]. This process reflects a complex thromboinflammatory mechanism driven not only by activation of the coagulation cascade but also by the interaction between platelet activation and the inflammatory response. During thrombus formation in PE, activated platelets contribute to thrombus stabilization and promote vascular inflammation through the release of inflammatory mediators. Accordingly, biomarkers associated with platelet activation have been increasingly investigated in recent years as potential indicators of disease severity and clinical outcomes in PE [3–5]. GPVI is a receptor expressed on the platelet surface that plays a central role in collagen‐mediated platelet activation and is critically involved in platelet adhesion, aggregation, and thrombus stabilization [6–9]. Recent experimental work indicates that GPVI signaling can vary with platelet production state, emphasizing the context dependence of GPVI biology [13]. Owing to these biological properties, alterations in GPVI levels may be associated with thrombus burden and platelet activation, which in turn may be linked to clinical severity in PE.

Conventional markers in PE reflect partially different biological domains, including fibrin turnover, myocardial injury or strain, systemic inflammation, and tissue hypoperfusion [3–5, 14–19]. Contemporary risk‐stratification approaches increasingly integrate several of these signals with clinical variables, including in multimarker models [20, 21]. In the present dataset, GPVI did not correlate significantly with D‐dimer, troponin, NLR, SII, or lactate. Rather than supporting the earlier claim that GPVI simply tracks thrombotic or inflammatory burden, this lack of correlation suggests that circulating GPVI may capture a biological dimension not directly represented by these routinely used markers. This interpretation remains exploratory and requires mechanistic and external clinical validation.

Platelet activation and platelet receptors are among the key mechanisms involved in thrombus formation and stabilization. In particular, GPVI, a receptor expressed on the platelet surface, plays a critical role in collagen‐mediated platelet activation and thrombus stabilization [6–9]. Experimental studies have shown that GPVI‐mediated signaling pathways are essential for thrombus growth and stability, and that the interaction between fibrin and GPVI may enhance platelet procoagulant activity [7]. Similarly, the role of GPVI in thrombus progression and stability has been demonstrated in both experimental and translational studies [6]. In addition, GPVI‐mediated platelet activation, together with integrin‐dependent adhesion mechanisms, has been reported to represent a key regulatory checkpoint in thrombus formation [8]. The role of platelet activation in the pathophysiology of venous thrombosis has gained increasing attention, and GPVI has been proposed as an important platelet receptor, particularly in the development of venous thrombotic complications [9].

The study‐recorded ordinal CTPA categories were defined in the original case‐report form by anatomical level: pulmonary artery embolism (higher radiological burden), pulmonary artery branch embolism (intermediate burden), and a single pulmonary arteriole embolism (lower burden). We retained these categories as descriptive radiological strata but removed the original mild/moderate/severe terminology because it could be confused with guideline‐defined clinical risk. CTPA‐based clot‐burden quantification has established precedent, including the Qanadli obstruction index [12], but our three‐level legacy CRF classification is not equivalent to a validated obstruction score. A separate exploratory ESC‐aligned analysis based on PESI, RV dysfunction, and troponin was therefore added. GPVI differed across those reconstructed categories (*p* = 0.045), but intermediate‐low and intermediate‐high medians were similar and the extreme categories contained few patients; this result should be viewed as supportive rather than definitive.

The diagnostic component requires particular caution. GPVI had a higher AUC than D‐dimer in the comparison of CTPA‐confirmed PE with control participants, but this design does not reproduce the emergency‐department diagnostic problem of distinguishing PE from symptomatic patients in whom PE is excluded. The very different biomarker distributions between patients with PE and control participants in whom PE was not clinically suspected can inflate discrimination through spectrum effects. Accordingly, we do not interpret the AUC of 0.968 as evidence that GPVI is ready for diagnostic use, and no claim is made that GPVI outperforms established pretest‐probability/D‐dimer pathways. A prospective study enrolling consecutive patients with suspected PE and including a CTPA‐negative comparator is required to determine clinically relevant diagnostic accuracy and incremental value.

Clinically meaningful longitudinal outcomes such as in‐hospital mortality, 30‐day mortality, hemodynamic deterioration, and reperfusion therapy requirement were not collected in a sufficiently standardized prospective manner for validated outcome analysis. ICU admission was therefore used only as an available severity‐related clinical endpoint. This limitation is important because risk‐stratification biomarkers should ultimately be evaluated against hard outcomes in longitudinal cohorts.

The incremental ICU analysis also provides context for comparison with established multivariable risk approaches. Contemporary PE risk stratification increasingly integrates clinical scores, cardiac biomarkers, and multimarker models [20, 21]. Artac et al. reported that the heart failure–derived MAGGIC score carried prognostic information in 302 patients with acute PE, illustrating that composite models integrating comorbidity and physiological variables may generalize beyond their original disease context [22]. MAGGIC and GPVI‐based assessment are conceptually different: MAGGIC summarizes multidimensional clinical risk, whereas GPVI is a single platelet‐related biomarker. In our modest cohort, adding GPVI to PESI increased apparent discrimination, but performance attenuated with internal validation and the large exploratory NRI/IDI estimates should not be overinterpreted. Whether GPVI improves hard‐outcome prediction beyond validated PE‐specific models remains unresolved.

Notably, the apparent AUC of the combined PESI + GPVI model (0.968) was essentially identical to that of GPVI alone (0.969). Thus, although adding GPVI improved discrimination compared with PESI alone in this dataset, the combined model did not demonstrate additional discrimination beyond GPVI alone. This suggests that the observed improvement was predominantly attributable to GPVI rather than to complementary information from the two predictors and further supports a cautious, exploratory interpretation pending external validation.

### 4.1. Study Limitations

This study has several limitations. First, the single‐center design and modest sample size limit precision and generalizability, and no formal a priori sample‐size calculation was performed. Second, the comparator group consisted of emergency department patients presenting with other acute complaints in whom PE was not clinically suspected, rather than symptomatic patients with suspected PE in whom PE was excluded by CTPA. Two consequences follow. First, the case–control design is subject to spectrum bias and almost certainly overestimates discrimination relative to the real‐world diagnostic task, so the reported AUC cannot be interpreted as diagnostic accuracy. Second, because CTPA was not performed in the control group, PE was not formally excluded by the reference standard, and clinically silent PE cannot be entirely ruled out in these participants. Third, the cross‐sectional design and single‐time‐point GPVI measurement preclude causal inference and temporal assessment. Fourth, the study‐recorded three‐level CTPA classification was a legacy anatomical descriptor rather than a validated clinical risk score; the added ESC‐aligned analysis was reconstructed from available baseline variables, and admission systolic blood pressure < 90 mmHg was only a proxy for possible high‐risk PE because persistence of hypotension, obstructive shock, vasopressor requirement, and cardiac arrest were not systematically captured. Fifth, symptom‐onset‐to‐sampling time and the timing of sampling relative to anticoagulant administration were not prospectively recorded. Sixth, study‐specific intra‐ and inter‐assay precision, specimen‐level duplicate results, assay lot/batch information, freeze–thaw counts, and laboratory blinding records were not available in the records reviewed for this revision and therefore cannot be reported retrospectively. Seventh, GPVI was measured in serum rather than anticoagulated plasma. Because ex vivo coagulation during serum preparation can induce GPVI ectodomain shedding [23], the absolute concentrations observed in this study may partly reflect the sample matrix and processing conditions and may not be directly comparable with plasma sGPVI concentrations reported in other cohorts. Although samples were handled according to a common study protocol, reducing differential preanalytical variation within the cohort, systematic matrix‐related effects cannot be excluded. Assay‐specific stability under the applied storage conditions was not formally evaluated. Future studies should use standardized preanalytical procedures and, ideally, compare matched serum and plasma samples. Eighth, standardized central/lobar/segmental/subsegmental location labels were not available for reliable post hoc redistribution beyond the study‐recorded categories. Ninth, hard longitudinal outcomes, including 30‐day mortality and hemodynamic deterioration, were not systematically available for validated prognostic analysis, and ICU admission may be influenced by local practice. Finally, the high apparent ICU discrimination and reclassification estimates may be optimistic despite internal validation. The percentile interval for category‐free NRI reached its theoretical upper bound of 2.000, further indicating boundary instability in this modest cohort. External validation in larger multicenter cohorts is essential.

## 5. Conclusion

Serum GPVI was associated with radiological thrombus burden and severity‐related clinical indicators in acute PE. Although GPVI showed apparent discrimination between patients with PE and control participants, the case–control comparator design does not establish real‐world diagnostic accuracy. These findings should therefore be considered exploratory and require confirmation in larger prospective cohorts that include symptomatic CTPA‐negative comparators and hard clinical outcomes.

## Author Contributions

Conceptualization, Mert Metin İnan and Mustafa Burak Sayhan; methodology, Mustafa Burak Sayhan, Eray Çeliktürk, and Esin Seçgin Sayhan; investigation, Mert Metin İnan and Sinem Çalın; data curation, Mert Metin İnan; formal analysis, Mert Metin İnan and Esin Seçgin Sayhan; validation, Ömer Salt and Eray Çeliktürk; visualization, Esin Seçgin Sayhan; supervision, Mustafa Burak Sayhan and Ömer Salt; project administration, Mustafa Burak Sayhan; resources, Mert Metin İnan and Eray Çeliktürk; writing–original draft, Mert Metin İnan; and writing–review and editing, Mustafa Burak Sayhan, Ömer Salt, and Eray Çeliktürk.

## Funding

This research received no specific grant from any funding agency in the public, commercial, or not‐for‐profit sectors.

## Disclosure

All authors have read and approved the final version of the manuscript. The corresponding author, acting as manuscript guarantor, had full access to all of the data in this study and takes complete responsibility for the integrity of the data and the accuracy of the data analysis. The corresponding author, acting as manuscript guarantor, affirms that this manuscript is an honest, accurate, and transparent account of the study being reported; that no important aspects of the study have been omitted; and that any discrepancies from the study as originally described have been explained in the revision. No funding source or financial relationship had any role in study design; data collection, analysis, or interpretation; preparation of the manuscript; or the decision to submit the work for publication.

## Conflicts of Interest

The authors declare no conflicts of interest.

## Supporting Information

Additional supporting information can be found online in the Supporting Information section.

## Supporting information


**Supporting Information** STROBE‐checklist.

## Data Availability

The data supporting the findings of this study are available from the corresponding author upon reasonable request. The data are not publicly available due to privacy and ethical restrictions related to patient information.
