# Supplementary material for: Serum Glycoprotein VI in Acute Pulmonary Embolism: Association With Radiological Thrombus Burden and Clinical Risk Indicators
Source: Can Respir J. 2026 Oct 18;2026:9286221. doi: 10.1155/carj/9286221 (PMC13677138; doi:10.1155/carj/9286221)
Supplement: Supplementary file 1 [file CARJ-2026-9286221-s001.docx]

STROBE Statement

|  | Item No. | Recommendation | Page  No. | Relevant text from manuscript |
| --- | --- | --- | --- | --- |
| **Title and abstract** | 1 | (*a*) Indicate the study’s design with a commonly used term in the title or the abstract | 1-2 | Prospective observational study design is stated in the Methods section of the abstract. |
|  |  | (*b*) Provide in the abstract an informative and balanced summary of what was done and what was found | 1-2 | The abstract provides a structured and balanced summary including Background, Objective, Methods, Results, and Conclusion. |
| Introduction | | | |  |
| Background/rationale | 2 | Explain the scientific background and rationale for the investigation being reported | 3-4 | Scientific background describing thromboinflammatory mechanisms and the role of GPVI is presented in the Introduction. |
| Objectives | 3 | State specific objectives, including any prespecified hypotheses | 4 | The objective is clearly stated in the final paragraph of the Introduction. |
| Methods | | | |  |
| Study design | 4 | Present key elements of study design early in the paper | 5 | A prospective, single-center observational study design is described. |
| Setting | 5 | Describe the setting, locations, and relevant dates, including periods of recruitment, exposure, follow-up, and data collection | 5 | The study was conducted in the Emergency Department between January 2023 and June 2023. |
| Participants | 6 | (*a*) *Cohort study*—Give the eligibility criteria, and the sources and methods of selection of participants. Describe methods of follow-up  *Case-control study*—Give the eligibility criteria, and the sources and methods of case ascertainment and control selection. Give the rationale for the choice of cases and controls  *Cross-sectional study*—Give the eligibility criteria, and the sources and methods of selection of participants | 5-6 | Eligibility criteria, inclusion and exclusion criteria, and participant selection are described. |
|  |  | (*b*) *Cohort study*—For matched studies, give matching criteria and number of exposed and unexposed  *Case-control study*—For matched studies, give matching criteria and the number of controls per case | NA | Not a matched study |
| Variables | 7 | Clearly define all outcomes, exposures, predictors, potential confounders, and effect modifiers. Give diagnostic criteria, if applicable | 6-7 | All variables (GPVI, D-dimer, troponin, NLR, SII, lactate, PBTA, qSOFA, PESI, ICU) are defined. |
| Data sources/ measurement | 8* | For each variable of interest, give sources of data and details of methods of assessment (measurement). Describe comparability of assessment methods if there is more than one group | *6-7* | Laboratory and clinical measurement methods are described. |
| Bias | 9 | Describe any efforts to address potential sources of bias | 7 | Standardized data collection and laboratory procedures were used to minimize bias. |
| Study size | 10 | Explain how the study size was arrived at | 5 | The study included 75 participants based on available eligible patients. |

Continued on next page

| Quantitative variables | 11 | Explain how quantitative variables were handled in the analyses. If applicable, describe which groupings were chosen and why | 7-8 | Continuous variables were expressed as median (IQR); clinical classifications were predefined. | |
| --- | --- | --- | --- | --- | --- |
| Statistical methods | 12 | (*a*) Describe all statistical methods, including those used to control for confounding | 7-8 | Statistical tests including Mann–Whitney U, Kruskal–Wallis, chi-square, Spearman correlation, ROC, and logistic regression are described. | |
|  |  | (*b*) Describe any methods used to examine subgroups and interactions | 8-9 | Subgroup analyses based on severity and clinical risk indicators were performed. | |
|  |  | (*c*) Explain how missing data were addressed | NA | No missing data reported | |
|  |  | (*d*) *Cohort study*—If applicable, explain how loss to follow-up was addressed  *Case-control study*—If applicable, explain how matching of cases and controls was addressed  *Cross-sectional study*—If applicable, describe analytical methods taking account of sampling strategy | NA | Not applicable for cross-sectional observational analysis | |
|  |  | (*e*) Describe any sensitivity analyses | NA | No sensitivity analysis performed. | |
| Results | | | | |  |
| Participants | 13* | (a) Report numbers of individuals at each stage of study—eg numbers potentially eligible, examined for eligibility, confirmed eligible, included in the study, completing follow-up, and analysed | 9 | A total of 75 participants were included. | |
|  |  | (b) Give reasons for non-participation at each stage | NA | No exclusions after enrollment. | |
|  |  | (c) Consider use of a flow diagram | NA | Flow diagram not included. | |
| Descriptive data | 14* | (a) Give characteristics of study participants (eg demographic, clinical, social) and information on exposures and potential confounders | 9-10 | Baseline characteristics are presented in Table 1. | |
|  |  | (b) Indicate number of participants with missing data for each variable of interest | NA | No missing data reported | |
|  |  | (c) *Cohort study*—Summarise follow-up time (eg, average and total amount) | NA | Not applicable | |
| Outcome data | 15* | *Cohort study*—Report numbers of outcome events or summary measures over time | 10-13 | Outcome results are presented in Tables 2–6. | |
|  |  | *Case-control study—*Report numbers in each exposure category, or summary measures of exposure |  |  | |
|  |  | *Cross-sectional study—*Report numbers of outcome events or summary measures |  |  | |
| Main results | 16 | (*a*) Give unadjusted estimates and, if applicable, confounder-adjusted estimates and their precision (eg, 95% confidence interval). Make clear which confounders were adjusted for and why they were included | 10-13 | AUC values and ORs with 95% CI are reported. | |
|  |  | (*b*) Report category boundaries when continuous variables were categorized | 12-13 | Cut-off values are defined. | |
|  |  | (*c*) If relevant, consider translating estimates of relative risk into absolute risk for a meaningful time period | NA | Not applicable | |

Continued on next page

| Other analyses | 17 | Report other analyses done—eg analyses of subgroups and interactions, and sensitivity analyses | 11-13 | Correlation and subgroup analyses are reported. |
| --- | --- | --- | --- | --- |
| Discussion | | | | |
| Key results | 18 | Summarise key results with reference to study objectives | 14 | Key findings summarized. |
| Limitations | 19 | Discuss limitations of the study, taking into account sources of potential bias or imprecision. Discuss both direction and magnitude of any potential bias | 17 | Limitations discussed in Section 4.1. |
| Interpretation | 20 | Give a cautious overall interpretation of results considering objectives, limitations, multiplicity of analyses, results from similar studies, and other relevant evidence | 14-17 | Results interpreted in context. |
| Generalisability | 21 | Discuss the generalisability (external validity) of the study results | 17 | External validity discussed. |
| Other information | |  | | |
| Funding | 22 | Give the source of funding and the role of the funders for the present study and, if applicable, for the original study on which the present article is based | 18 | No external funding. |

*Give information separately for cases and controls in case-control studies and, if applicable, for exposed and unexposed groups in cohort and cross-sectional studies.

**Note:** An Explanation and Elaboration article discusses each checklist item and gives methodological background and published examples of transparent reporting. The STROBE checklist is best used in conjunction with this article (freely available on the Web sites of PLoS Medicine at http://www.plosmedicine.org/, Annals of Internal Medicine at http://www.annals.org/, and Epidemiology at http://www.epidem.com/). Information on the STROBE Initiative is available at www.strobe-statement.org.
